# Predictive Value of CFIm25 Expression in Peripheral Blood Monocytes for Coronary Atherosclerosis

**DOI:** 10.7150/ijms.91148

**Published:** 2024-01-20

**Authors:** Meng Liu, Xiaoxiao Chen, Zhan Gu, Hao He, Mingyue Chen, Lin Kuai, Zhiqiang Jia, Yi Li, Yizhou Chen, Mei Hong, Fangping Xiao

**Affiliations:** Department of Cardiology, The Second Affiliated Hospital of Nanjing Medical University, Nanjing, 210011, China.

**Keywords:** cleavage factor Im25, coronary atherosclerosis, peripheral blood monocyte, Gensini score

## Abstract

**Background:** Cleavage factor Im25 (CFIm25) regulates cell function by affecting mRNA editing processes and plays diverse roles in various diseases. Studies have found that peripheral blood monocytes are valuable in diagnosing and prognosing coronary atherosclerosis. However, no studies have examined the predictive value of CFIm25 expression in peripheral blood monocytes for coronary atherosclerosis.

**Methods and Results:** We collected the coronary angiography results of 267 patients and calculated the Gensini score to evaluate their degree of coronary atherosclerosis. We isolated peripheral blood monocytes and detected CFIm25 RNA expression. Based on their Gensini score, we divided the patients into negative (0, *n* = 46), mild lesion (≤ 8, *n* = 71), moderate lesion (8-23, *n* = 76), and severe lesion (≥ 23, *n* = 74) groups. Results showed that CFIm25 expression correlated negatively with the Gensini score and the number of involved coronary vessels. Univariate and multivariate binary logistic regression analyses showed that CFIm25 expression in peripheral blood monocytes was a protective factor for severe lesions, ≥ 50% stenosis, and three-vessel lesions. The areas under the receiver operating characteristic curve of CFIm25 expression for predicting lesions, severe lesions, ≥50% stenosis, and three-vessel lesions were 0.743, 0.735, 0.791, and 0.736, respectively.

**Conclusions:** CFIm25 expression in peripheral blood monocytes correlates negatively with the degree of coronary atherosclerosis and helps predict the severity and number of coronary artery lesions.

## Introduction

The 25-kDa subunit of mammalian cleavage factor I (CFIm25) is a small protein encoded by the Nudix hydrolase 21 gene. The best-known role of CFIm25 is in the 3'-end processing of pre-mRNAs 1. Abnormalities in the 3'-end processing mechanism are common in many oncological, immunological, neurological, and hematological diseases and in cellular and molecular conditions important for cell homeostasis 2. Furthermore, lipopolysaccharide (LPS)-stimulated macrophages are accompanied by a shortening of the 3' untranslated region and a reduction in translation rate 3.

Atherosclerosis is characterized by chronic, non-resolving, low-grade, sterile arterial wall inflammation. Studies have shown that monocytes (MONOs) are important in atherosclerosis development and progression 4, 5. In the arterial wall, the accumulation of cholesterol lipoproteins leads to vascular endothelial damage, which then induces MONO recruitment to the damaged site and differentiation into macrophages. MONO recruitment during its early stages dominates lesion progression and is required for plaque expansion 6. Increased numbers of peripheral blood MONOs are found in patients with atherosclerosis and experimental animal models of atherosclerosis and correlate with plaque size and stage 7-10.

Based on the important role of peripheral blood MONOs in the process of coronary atherosclerosis and the possible correlation between CFIm25 and the physiological function of MONOs, this study aimed to detect the expression level of CFIm25 in peripheral blood MONOs and evaluate its predictive value for the severity of coronary atherosclerosis.

## Methods

### Ethics

The study protocol conforms to the ethical guidelines of the 1975 Declaration of Helsinki as reflected in a priori approval by the Medical Ethics Committee of the Second Affiliated Hospital of Nanjing Medical University (No. [2022]-KY188-01) 11. We also complied with the clinical trial registration statement from the International Committee of Medical Journal Editors (registration number: ChiCTR2200061732). Written informed consent was obtained from all patients. The patients' privacy was protected by hiding their names and other identifying information, with numbers representing each case.

### Patients

We enrolled 267 patients who were hospitalized in the Cardiovascular Center of the Second Affiliated Hospital of Nanjing Medical University between January and June 2022. The inclusion criteria were patients over 18 years old who were admitted to the hospital due to chest pain and underwent coronary angiography. We obtained coronary angiograms and calculated Gensini scores. We collected patients' peripheral blood and isolated their MONOs. The exclusion criteria included: (1) acute coronary syndrome; (2) previous medical history of coronary stents or bypass surgery due to myocardial infarction; (3) other severe cardiovascular diseases, including chronic heart failure (grade III-IV), myocarditis, cardiomyopathy, severe heart valve disease, myocardial bridge, pericardial effusion, pulmonary heart disease, and aortic dissection; (4) other major systemic diseases, including malignant tumors, systemic immune diseases, stress states (e.g., acute abdomen, cerebral hemorrhage, or acute phase of cerebral infarction), serious vital organ diseases (e.g., liver, kidney, pancreas, or thyroid), and acute or chronic infections.

### Data and sample collection

Detailed clinical data were collected, including demographic information, coronary atherosclerosis risk factors, and biochemical indicators. All data were obtained from the patients before coronary angiography at the same visit. We recorded each patient's age; sex; body mass index (BMI); smoking status; drinking status; hypertension history; diabetes history; neutrophil/lymphocyte ratio (NLR); white blood cell, neutrophil, lymphocyte, and MONO counts; international normalized ratio; and aspartate aminotransferase/alanine aminotransferase, albumin/globulin, blood urea nitrogen, total cholesterol (TC), total triglycerides (TG), high-density lipoprotein cholesterol, low-density lipoprotein cholesterol (LDL-C), and D-dimer levels. Approximately 10 mL of arterial blood was collected by cannulation through the radial artery before injection of contrast material and placed in a centrifuge tube containing ethylenediamine tetraacetic acid disodium for subsequent MONO isolation.

### Coronary atherosclerosis assessment

Gensini scores were calculated based on coronary angiography results. First, the coronary artery stenosis score was determined: 1 point for < 25%, 2 points for 25%-49%, 4 points for 50%-74%, 8 points for 75%-89%, 16 points for 90%-99%, and 32 points for 100% coronary artery stenosis. Second, the coefficient was determined according to the location of the coronary artery lesions: 5 for left main coronary artery lesions; 1.0, 2.5, and 1.5 for distal, proximal, and middle segments of the left anterior descending artery, respectively;1.0 and 0.5 for first and second diagonal branch lesions, respectively; 1.0 and 2.5 for the distal and proximal segments of the circumflex artery, respectively; 0 for lesions of the obtuse marginal branch and posterior descending branch; and 1.0 for lesions of the distal, proximal, and middle segments of the right coronary artery. The coronary lesion score = coronary stenosis score × corresponding lesion location coefficient. The Gensini score was the sum of all coronary lesion scores. The higher the Gensini score, the more severe the coronary atherosclerosis 12.

Based on the Gensini score, the patients were divided into the negative (0, *n* = 46) and coronary artery lesion groups (> 0, *n* = 221). Based on the tertiles of the Gensini score, the patients in the coronary artery lesion group were further divided into mild lesion (≤ 8, *n* = 71), moderate lesion (8-23 points, *n* = 76), and severe lesion (≥ 23 points, *n* = 74) groups 13.

Based on the American College of Cardiology and American Heart Association, ≥ 50% stenosis was diagnosed by coronary angiography based on the stenosis of ≥ 50% of the main branches of the subepicardial coronary artery (including left main coronary artery, left anterior descending artery, left circumflex artery, and right coronary artery) 14. A single-vessel lesion was defined as lumen stenosis of ≥ 50% in one major vessel. A double-vessel lesion was defined as lumen stenosis of ≥ 50% in two major vessels, including the left main coronary artery. A three-vessel lesion was defined as lumen stenosis of ≥ 50% in the three major vessels.

### Isolation and cultivation of peripheral blood MONOs

Approximately 10 mL of ethylenediaminetetraacetic acid-anticoagulated blood was transferred into a 15-mL centrifuge tube and centrifuged for 10 min (2000 r/min) to obtain precipitated cells. Cell suspensions were produced by diluting the precipitated cells 1:1 with phosphate-buffered saline (PBS). A volume of Ficoll lymphocyte separation solution equal to the cell suspension volume was added to another 15-mL centrifuge tube. The cell suspension was slowly added along the wall of the centrifuge tube 1 cm above the separation solution so that it lay on top before centrifugation for 25 min (2200 r/min). The upper layer was liquid (PBS), the middle layer was tunica albuginea (MONOs and lymphocytes), the lower layer was liquid phase 2 (Ficoll lymphocyte separation solution), and the lowest layer was the cell layer (other blood cells and residual tissue). The intermediate tunica albuginea layer was carefully aspirated into a new centrifuge tube, 3-4 volumes of PBS were added, and the tube was centrifuged for 10 min (1500 r/min). Next, the pellet was washed twice with PBS. Then, the cells were cultured in RPMI-1640 medium for 24 hours, and the adherent cells (MONOs) were collected to extract mRNA for CFIm25 expression quantification 15. This purification method resulted in MONOs with a purity of >90% 16.

### Extraction and real-time quantitative PCR of CFIm25

We extracted the RNAs from cells with TRIzol Reagent and then reverse-transcribed them into cDNA using a reverse transcription kit (Vazyme Biotech, Nanjing, China). Then, we performed real-time quantitative PCR to measure CFIm25 expression, which was normalized to glyceraldehyde-3-phosphate dehydrogenase (GAPDH) expression and calculated using the 2^-ΔΔCt^ method. The primer sequences used were: forward primer for CFIm25 (GGTCACTCAGTTCGGCAACAA), reverse primer for CFIm25 (CTCATGCGCTGAAATCTGGC), forward primer for GAPDH (GGAGCCAAAAGGGTCATCACTC), and reverse primer for GAPDH (GAGGGGCCATCCACAGTCTTCT).

### Statistical analysis

The SPSS 25.0 software (IBM Corp., Armonk, NY, USA) was used for statistical analysis, and Prism 8.0 software (GraphPad Software, San Diego, CA, USA) was used to make visual scatterplots of the data. Count data were expressed as the number of cases (percentage) and compared between groups using the chi-square test. Normally distributed data were expressed as mean ± standard deviation and compared between two groups using the independent samples *t*-test and between the four groups using one-way analysis of variance. Non-normally distributed data were expressed as median (interquartile range) and compared between two groups using the Mann-Whitney U test and between the four groups using the Krusukal-Wallis test. Spearman correlation coefficient (*r*_s_) was used to evaluate correlations between variables. Univariate and multivariate binary logistic regression analysis was used to assess the correlation between CFIm25 expression and coronary atherosclerosis. The receiver operating characteristic (ROC) curve was used to assess the predictive value of CFIm25 expression for coronary atherosclerosis.

## Results

### Patients' characteristics

A total of 267 patients were enrolled in this study, including 46 patients in the negative group and 221 patients in the coronary artery lesion groups, including 71 patients in the mild lesion group, 76 patients in the moderate lesion group and 74 patients in the severe lesion group. There were 136 male patients and 131 female patients, including 20 males and 26 females in the negative group and 116 males and 105 females in the coronary artery lesion groups.

Table 1 shows significant differences in age, hypertension, diabetes, smoking, drinking, MONO count, urea nitrogen, D-dimer, and CFIm25 expression between the negative, mild lesion, moderate lesion, and severe lesion groups (*P* < 0.05). CFIm25 expression in the negative group was lower than that in the other three groups with coronary artery lesions.

### Comparison of variables between groups

**Figure 1:**
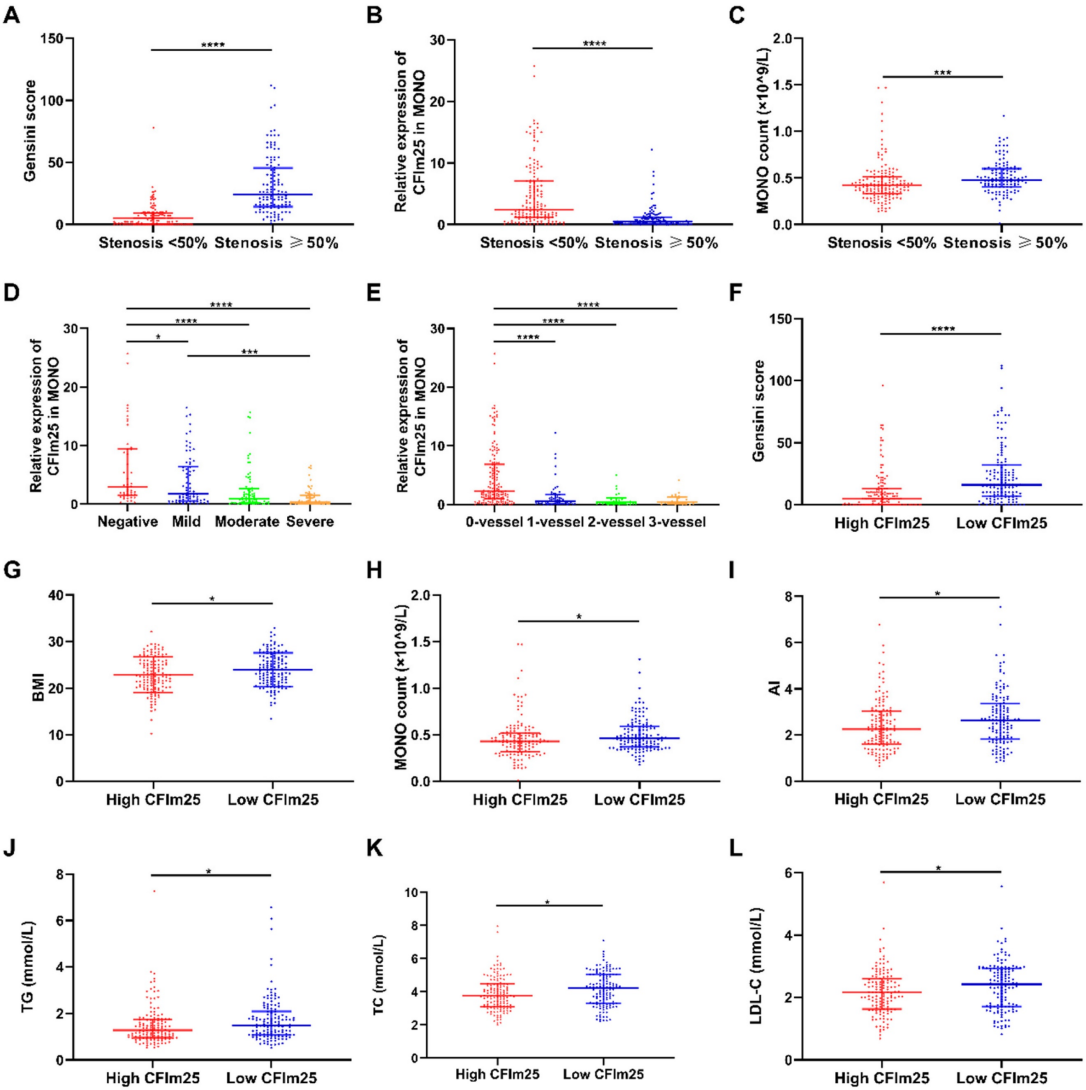
** Comparison of variables between groups.** (A-C) Variables between the <50% stenosis and ≥50% stenosis groups. (D) CFIm25 expression between the negative, mild, moderate, and severe lesion groups. (E) CFIm25 expression between the 0-vessel, single-vessel, two-vessel, and three-vessel lesion groups. (1F-L) Variables between high CFIm25 expression and low CFIm25 expression groups. CFIm25: Cleavage factor Im25; MONO: Monocyte; BMI: Body mass index; AI: Atherosclerosis index; TG: Total triglycerides; TC: Total cholesterol; LDL-C: Low-density lipoprotein cholesterol. *P < 0.05, ***P < 0.001, ****P < 0.0001.

Figures 1A-C compare the differences in variables between the < 50% stenosis and ≥ 50% stenosis groups. Gensini scores were higher in the stenosis ≥ 50% group (24 [14,46]) than in the stenosis < 50% group (5 [0,9]; *P* < 0.05; Figure 1A). CFIm25 expression was lower in the ≥ 50% stenosis group (0.45 [0.14, 1.22]) than in the < 50% stenosis group (2.44 [1.13, 7.12]; *P* < 0.05; Figure 1B). MONO counts were higher in the ≥ 50% stenosis group (0.48 [0.40, 0.60]; ×10^9^/L) than that in the < 50% stenosis group (0.42 [0.33, 0.51]; ×10^9^/L]; *P* < 0.05; Figure 1C).

Figure 1D compares the differences in CFIm25 expression between the negative, mild, moderate, and severe lesion groups. CFIm25 expression was higher in the negative group than in the three groups with coronary atherosclerosis (*P* < 0.05). While CFIm25 expression was higher in the mild group than in the severe group (*P* < 0.05), it did not differ significantly between the moderate and severe groups (*P* > 0.05). CFIm25 expression showed a continual downward trend from the negative group (2.95 [1.56, 9.48]) to the mild lesion group (1.74 [0.58, 6.44]) to the moderate lesion group (0.96 [0.26, 2.65]) to the severe lesion group (0.36 [0.11, 1.46]).

Figure 1E compares the CFIm25 expression differences between the 0-vessel, single-vessel, two-vessel, and three-vessel lesion groups. CFIm25 expression was lower in the 0-vessel lesion group than in the three groups with diseased vessels (*P* < 0.05). CFIm25 expression did not differ significantly among the single-vessel, double-vessel, and three-vessel lesion groups (*P* > 0.05). However, CFIm25 expression showed a decreasing trend as the number of diseased vessels increased, from 2.32 (0.98, 6.90) in the 0-vessel lesion group to 0.63 (0.18, 1.67) in the single-vessel lesion group to 0.28 (0.11, 1.08) in the double-vessel lesion group to 0.41 (0.11, 0.99) in the three-vessel lesion group.

Figures 1F-L compare the differences in variables between high CFIm25 expression (the 50% of patients with higher CFIm25 expression) and low CFIm25 expression (the 50% of patients with lower CFIm25 expression) groups. Gensini score, BMI, MONO count, atherosclerosis index, TG, TC, and LDL-C were higher in the low CFIm25 expression group than in the high CFIm25 expression group (*P* < 0.05). Gensini scores were higher in the low CFIm25 expression group (16 [7, 32]) than in the high CFIm25 expression group (5 [0, 13]; *P* < 0.05; Figure 1F). BMIs were higher in the low CFIm25 expression group (24.00 ± 3.57 kg/m^2^) than in the high CFIm25 expression group (22.86 ± 3.83 kg/m^2^; *P* < 0.05; Figure 1G). MONO counts were higher in the low CFIm25 expression group (0.46 [0.37, 0.59]; ×10^9^/L) than in the high CFIm25 expression group (0.43 [0.32, 0.52]; ×10^9^/L; *P* < 0.05; Figure 1H). Atherosclerosis indices were higher in the low CFIm25 expression group (2.64 [1.84, 3.38]) than in the high CFIm25 expression group (2.25 [1.59, 3.03]; *P* < 0.05; Figure 1I). TG levels were higher in the low CFIm25 expression group (1.50 [1.07, 2.09]; mmol/L) than in the high CFIm25 expression group (1.28 [0.96, 1.75]; mmol/L; *P* < 0.05; Figure 1J). TC levels were higher in the low CFIm25 expression group (4.23 [3.30, 5.05]; mmol/L) than in the high CFIm25 expression group (3.75 [3.12, 4.48]; mmol/L; *P* < 0.05; Figure 1K). LDL-C levels were higher in the low CFIm25 expression group (2.43 [1.71, 2.93]; mmol/L) than in the high CFIm25 expression group (2.16 [1.63, 2.59]; mmol/L; *P* < 0.05; Figure 1L).

### Correlation analysis between CFIm25 expression in peripheral blood MONOs and related variables

**Figure 2:**
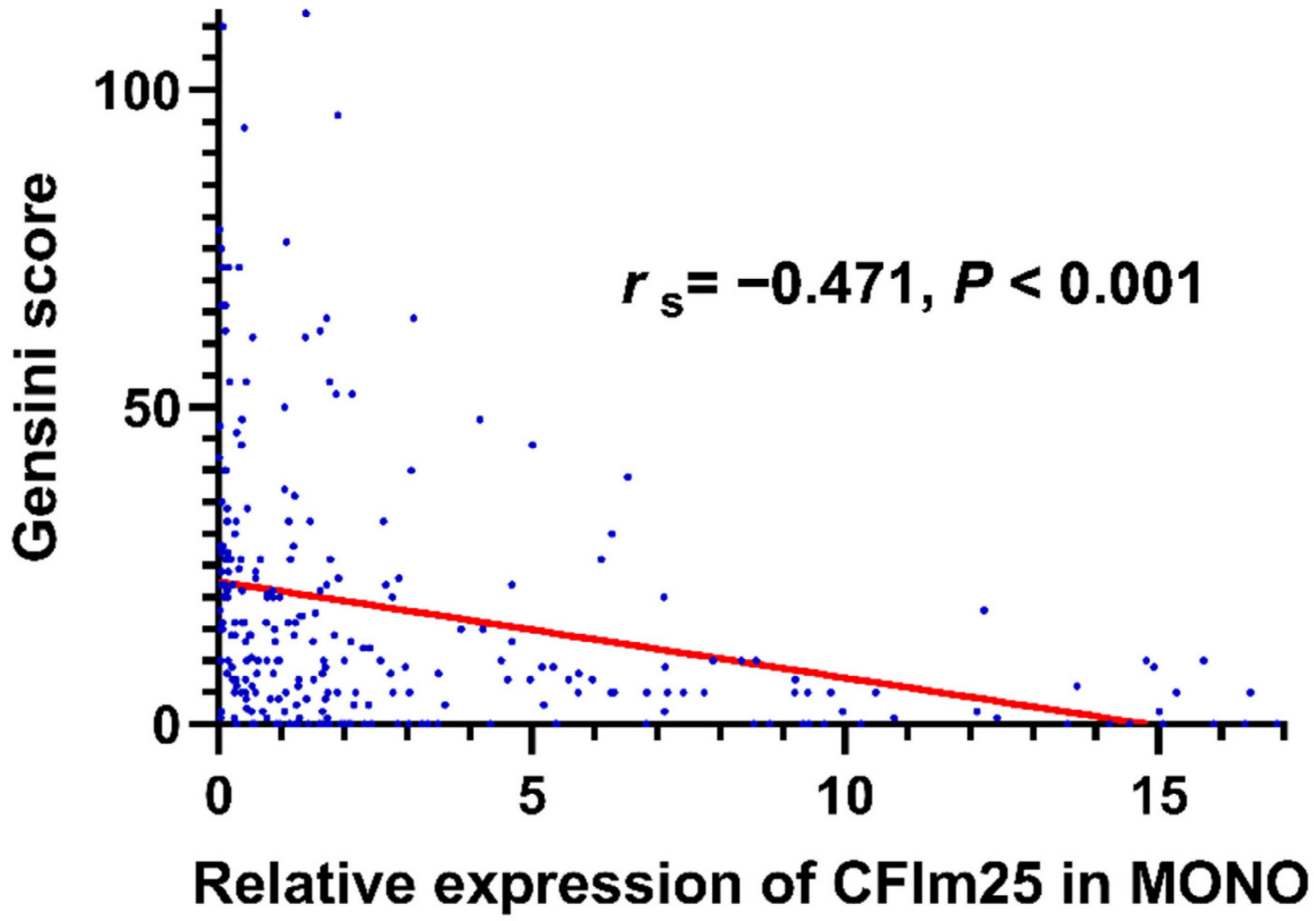
Correlation analysis between CFIm25 expression in peripheral blood MONOs and Gensini scores.

Spearman correlation analysis showed that CFIm25 expression correlated negatively with the Gensini score (*r*_s_ = -0.471, *P* < 0.001; Figure 2) and the number of involved branches (*r*_s_ = -0.486, *P* < 0.001). Table 2 lists the correlations of CFIm25 expression with other related variables. CFIm25 expression was weakly negatively correlated with BMI, atherosclerosis index, TC, TG, LDL-C, and D-dimer and weakly positively correlated with prealbumin. After adjusting for these variables, CFIm25 expression remained negatively correlated with the Gensini score (*r*_s_ = -0.256, *P* < 0.001) and the number of involved branches (*r*_s_ = -0.315, *P* < 0.001).

### Univariate and multivariate regression analyses of CFIm25 expression in peripheral blood MONOs

In the univariate binary logistic regression analysis, CFIm25 expression in peripheral blood MONOs was a protective factor for severe lesions (odds ratio [OR] = 0.624, 95% confidence interval [CI]: 0.531-0.732, *P* < 0.05), stenosis ≥ 50% (OR = 0.775, 95% CI: 0.706-0.852, *P* < 0.05), and three-vessel lesions (OR = 0.168, 95% CI: 0.097-0.290, *P* < 0.05). In a multivariate binary logistic regression analysis adjusting for coronary atherosclerosis risk factors, including sex, age, hypertension, smoking, diabetes, TC, TG, and LDL-C, CFIm25 expression in peripheral blood MONOs was a protective factor for severe lesions (OR = 0.683, 95% CI: 0.566-0.823, *P* < 0.05), stenosis ≥ 50% (OR = 0.650, 95% CI: 0.561-0.755, *P* < 0.05), and three-vessel lesions (OR = 0.488, 95% CI: 0.291-0.820, *P* < 0.05; Table 3).

### Predictive value of CFIm25 expression in peripheral blood MONOs for coronary artery lesions

**Figure 3:**
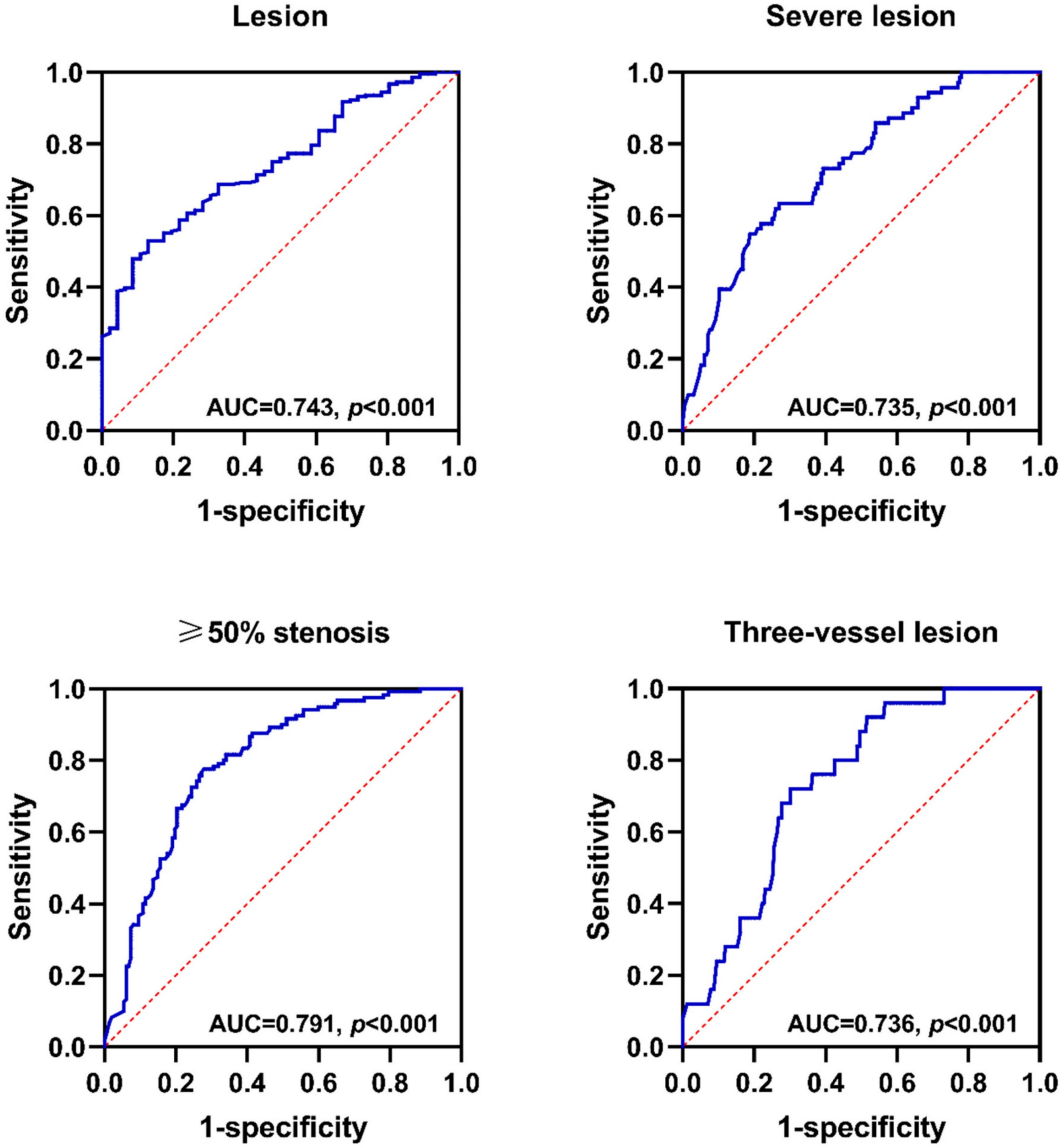
** Predictive value of CFIm25 expression in peripheral blood MONOs for coronary atherosclerosis lesions.** (A) ROC curve analyses of CFIm25 expression for lesions. (B) ROC curve analyses of CFIm25 expression for severe lesions. (C) ROC curve analyses of CFIm25 expression for ≥50% stenosis. (D) ROC curve analyses of CFIm25 expression for three-vessel lesions. AUC: Area under the curve.

A ROC curve was used to assess the predictive value of CFIm25 expression in peripheral blood MONOs for coronary atherosclerosis. Figure 3 shows that CFIm25 expression had predictive value for coronary atherosclerosis lesions, severe lesions, ≥ 50% stenosis, and three-vessel lesions. When the expression of CFIm25 had a cut-off value of 0.399, the predictive efficiency on lesions was the highest, with a sensitivity of 0.870, a specificity of 0.529, and an area under the curve (AUC) of 0.743 (*P* < 0.001). When the expression of CFIm25 had a cut-off value of 0.365, the predictive efficiency on severe lesions was the highest, with a sensitivity of 0.745, a specificity of 0.620, and an AUC of 0.735 (*P* < 0.001). When the expression of CFIm25 had a cut-off value of 0.503, the predictive efficiency on ≥ 50% stenosis was the highest, with a sensitivity of 0.728, a specificity of 0.775, and an AUC of 0.791 (*P* < 0.001). When the expression of CFIm25 had a cut-off value of 0.418, the predictive efficiency on three-vessel lesions was the highest, with a sensitivity of 0.698, a specificity of 0.720, and an AUC of 0.736 (*P* < 0.001).

## Discussion

In our study, when patients were divided into four groups with different degrees of coronary atherosclerosis based on their Gensini score, CFIm25 expression in peripheral blood MONOs was higher in the negative group than in the three groups with coronary atherosclerosis and in the mild group than in the severe group. These results suggest that CFIm25 expression is inversely correlated with coronary atherosclerosis severity, with lower CFIm25 expression associated with more severe lesions. There was no significant difference in CFIm25 expression between the mild and moderate lesion groups or between the moderate and severe lesion groups. However, CFIm25 expression showed an overall downward trend with increasing coronary atherosclerosis severity. The lack of statistical difference between groups may be due to the small variation between groups or the small sample size.

CFIm25 expression in peripheral blood MONOs was lower in the ≥ 50% stenosis group than in the < 50% stenosis group and showed a decreasing trend with increases in the number of vessels with ≥ 50% stenosis. CFIm25 expression was significantly lower in the 0-vessel lesion group than in the single-vessel, double-vessel, or three-vessel lesion groups. CFIm25 expression did not differ significantly among single-vessel, double-vessel, and three-vessel lesion groups, suggesting that CFIm25 expression in peripheral blood MONOs may play an important role in promoting the occurrence and development of ≥ 50% coronary artery stenosis.

In addition, when grouping patients according to their CFIm25 expression, those in the low expression group had higher Gensini scores and more vascular involvement than those in the high expression group, suggesting that reduced CFIm25 expression in peripheral blood MONOs accelerates coronary atherosclerosis progression, including the expansion of the lesion range and the aggravation of stenosis.

Further correlation analysis showed that CFIm25 expression correlated negatively with the Gensini score and the number of involved vessels. Univariate and multivariate binary logistic regression analyses showed that CFIm25 expression was associated with severe lesions, ≥50% stenosis, and three-vessel lesions. Notably, it was beneficial in reducing the risks for the above lesions, especially the risk of three-vessel lesions. ROC curve analysis showed that CFIm25 expression in peripheral blood MONOs had predictive value for coronary atherosclerosis severe lesions, ≥50% stenosis, and three-vessel lesions.

These results suggest that we may have discovered a new coronary atherosclerosis-related pathogenic molecule that can be detected with a small amount of peripheral blood, time, and cost and has great predictive value for coronary atherosclerosis, especially serious lesions. It may provide new therapeutic targets and methods for preventing and treating coronary atherosclerosis in the future. In addition, lipid metabolism-related indicators, including the atherosclerosis index, TG, TC, and LDL-C, were higher in the low CFIm25 expression group than in the high CFIm25 expression group; correlation analysis showed that CFIm25 expression was weakly negatively correlated with these indicators. Therefore, the role of CFIm25 in coronary atherosclerosis may be related to lipid metabolism.

In our study, when we grouped patients according to their degree of coronary atherosclerotic lesions, MONO counts tended to increase with the degree of lesions. In addition, patients in the ≥50% stenosis group had higher MONO counts than those in the <50% stenosis group. Many studies have found increased MONO counts in the peripheral blood of patients with coronary atherosclerosis 17-19. Interestingly, MONOs are in a homeostatic state in the blood, bone marrow, and spleen 20, 21. These short-lived cells do not proliferate in the blood 22. However, they have a potential role in the renewal of tissue macrophages 23. Since the increase in MONO counts in patients with coronary atherosclerosis is unrelated to their proliferation, we speculate that the increase in MONO counts may be related to changes in life activities such as differentiation.

Maiwald et al. retrospectively reviewed the current literature on MONO gene expression and its association with coronary atherosclerosis. They identified genetic differences in peripheral blood MONOs between patients with and without coronary atherosclerosis, which could contribute to our understanding of the pathophysiology of coronary atherosclerosis. However, due to small sample sizes, isolation methods, and differences in the clinical phenotypes of included patients, previous studies have not consistently reported differentially expressed genes 24.

LPS-treated macrophage cell line RAW 264.7 is commonly used in basic experiments to construct a cell model of inflammation and oxidative stress, which can be used to simulate macrophages during the occurrence and development of atherosclerosis *in vivo* 25. The gene profile changes observed in LPS-stimulated macrophages were found to be caused by the 3'-end processing mechanism of mRNA, and the expression level of a single mRNA processing factor could affect the expression of multiple genes 3. LPS can specifically stimulate macrophages to produce tumor necrosis factor (TNF). The decrease in translation efficiency of *TNF* mRNA was related to its 3' untranslated region, and the regulation of its biosynthesis may occur at the translation level 26. Another study found that LPS stimulation of macrophages induced *TNF* transcription, accumulation of *TNF* mRNA in the cytoplasm, TNF binding to polyribosomes, and increased TNF protein secretion. This process was accompanied by a change in apparent *TNF* mRNA length, which might reflect the presence or absence of the 3' poly (A) tail 27.

Cathepsin S (CTSS) mRNA encodes a cysteine protease associated with angiogenesis and atherosclerosis 28. Treating cells with hypoxia or inflammatory cytokines induces editing of the 3'-end of the CTSS mRNA, increasing CTSS expression 29. These findings suggest that the 3'-end editing of mRNAs of key factors in macrophages accompanies the occurrence and development of atherosclerosis. However, no studies have examined CFIm25 expression in peripheral blood MONOs, and its effect and predictive value for coronary atherosclerosis have not been reported. The function and molecular mechanism of CFIm25 in MONOs require further exploration.

However, this study had several limitations. The validity of our experiments may have been limited by the small volume of peripheral blood collected and the small number of MONOs extracted to minimize harm to the patients, which may have led to some errors in our findings. In addition, limited by the small amount of blood collected and experimental conditions, only one method was used to detect CFIm25 expression in this study. Further studies are needed to expand the sample population and use more diverse detection methods.

## Conclusions

CFIm25 expression in peripheral blood MONOs is associated with coronary atherosclerosis severity and helps to predict coronary atherosclerosis severity and the number of diseased vessels. Therefore, it may be a new marker for coronary atherosclerosis.

## Figures and Tables

**Table 1:** Patients' characteristics.

Variable	Negative group (0, *n* = 46)	Mild lesion group (≤8, *n* = 71)	Moderate lesion group (8-23 points, *n* = 76)	Severe lesion group (≥23 points, *n* = 74)	Statistic	*P-value*
**Age (years)**	60.28 ± 13.64	65.00 ± 12.16	65.72 ± 12.75	67.94 ± 12.07	3.524	0.016
**BMI (kg/m^2^)**	23.83 ± 3.64	22.86 ± 3.85	22.90 ± 3.84	24.26 ± 3.42	2.464	0.063
**Sex, male (%)**	20 (43.5)	42 (56.8)	37 (48.7)	37 (52.1)	2.220	0.528
**Hypertension, case (%)**	32 (69.6)	36 (48.6)	55 (72.4)	55 (77.5)	15.822	0.001
**Diabetes, case (%)**	9 (19.6)	8 (10.8)	24 (31.6)	30 (42.3)	20.494	<0.001
**Smoking, case (%)**	8 (17.4)	6 (8.1)	8 (10.5)	23 (32.4)	18.456	<0.001
**Drinking, case (%)**	5 (10.9)	7 (9.5)	6 (7, 9)	16 (22.5)	8.597	0.035
**NLR**	1.94 (1.51, 2.82)	2.34 (1.71, 3.36)	2.56 (1.72, 3.65)	2.50 (2.05, 3.50)	7.469	0.058
**WBC count (×10^9^/L)**	5.63 (4.70, 6, 83)	6.07 (5.03, 7.09)	6.20 (5.26, 7.51)	6.07 (5.27, 7.48)	5.116	0.164
**NEUT count (×10^9^/L)**	3.27 (2.67, 4.27)	3.70 (3.01, 4.49)	3.88 (3.26, 4.80)	3.81 (3.24, 4.43)	7.138	0.068
**LYMP count (×10^9^/L)**	1.67 (1.23, 2.04)	1.55 (1.10, 2.12)	1.60 (1.13, 2.04)	1.45 (1.22, 1.87)	1.153	0.764
**MONO count (×10^9^/L)**	0.39 (0.30, 0.49)	0.44 (0.36, 0.56)	0.46 (0.38, 0.58)	0.47 (0.37, 0.60)	8.594	0.035
**AST/ALT**	1.01 (0.77, 1.38)	0.99 (0.86, 1.14)	1.08 (0.87, 1.41)	1.07 (0.92, 1.35)	4.151	0.246
**A/G**	1.63 (1.49, 1.96)	1.68 (1.52, 1.83)	1.63 (1.47, 1.81)	1.64 (1.42, 1.91)	0.892	0.827
**Urea (mmol/L)**	5.54 ± 1.70	6.44 ± 1.53	5.95 ± 1.69	6.46 ± 1.31	4.566	0.004
**TC (mmol/L)**	4.03 (3.43, 4.57)	3.83 (3.09, 4.59)	3.84 (3.14, 4.64)	4.32 (3.27, 5.37)	6.984	0.072
**TG (mmol/L)**	1.21 (1.03, 1.77)	1.40 (1.11, 1.80)	1.45 (1.00, 1.76)	1.51 (0.98, 2.26)	2.716	0.438
**HDL-C (mmol/L)**	1.17 (0.96, 1.38)	1.16 (1.03, 1.32)	1.17 (1.01, 1.33)	1.18 (0.95, 1.37)	0.134	0.987
**LDL-C (mmol/L)**	2.15 (1.70, 2.49)	2.16 (1.63, 2.67)	2.26 (1.73, 2.76)	2.58 (1.69, 2.99)	7.627	0.054
**INR**	1.01 (0.97, 1.04)	1.00 (0.96, 1.07)	1.01 (0.95, 1.07)	1.02 (0.93, 1.09)	0.259	0.968
**DD (ug/ml)**	0.57 (0.48, 0.70)	0.60 (0.48, 0.84)	0.62 (0.53, 0.77)	0.70 (0.57, 0.95)	11.465	0.009
**CFIm25 expression**	2.95 (1.56, 9.48)	1.74 (0.58, 6.44)	0.96 (0.26, 2.65)	0.36 (0.11, 1.46)	55.133	<0.001

BMI: Body mass index; NLR: Neutrophil/lymphocyte ratio; WBC: White blood cell; NEUT: Neutrophile; LYMP: Lymphocyte; MONO: Monocyte; AST/ALT: Glutamic oxaloacetic transaminase/glutamic pyruvic transaminase; A/G: Albumin/globulin; TC: Total cholesterol; TG: Total triglycerides; HDL-C: High-density lipoprotein cholesterol; LDL-C: Low-density lipoprotein cholesterol; INR: International normalized ratio; DD: D-dimer; CFIm25: Cleavage factor Im25.

**Table 2:** Correlation analysis between CFIm25 expression in peripheral blood MONOs and related variables.

Variable	*r*_s_	*P-value*
BMI (kg/m2)	-0.144	0.019
AI	-0.141	0.021
TC (mmol/L)	-0.173	0.005
TG (mmol/L)	-0.145	0.017
LDL-C (mmol/L)	-0.202	0.001
DD (ug/ml)	-0.162	0.008
PA (mg/dL)	0.124	0.042

BMI: Body mass index; AI: Atherosclerosis index; TC: Total cholesterol; TG: Total triglycerides; LDL-C: Low-density lipoprotein cholesterol; DD: D-dimer; PA: Prealbumin.

**Table 3:** Univariate and multivariate regression analyses of CFIm25 expression in peripheral blood MONOs.

	Univariate			Multivariate		
	OR	95% CI	*P-value*	OR	95% CI	*P-value*
Severe lesion	0.624	0.531-0.732	<0.001	0.683	0.566-0.823	<0.001
≥50% stenosis	0.775	0.706-0.852	0.004	0.650	0.561-0.755	<0.001
Three-vessel lesion	0.168	0.097-0.290	<0.001	0.488	0.291-0.820	0.007

OR: Odds ratio; CI: Credibility interval.

## Abbreviations

CFIm25: cleavage factor Im25
LPS: lipopolysaccharide
MONO: monocyte
BMI: body mass index
NLR: neutrophil/lymphocyte ratio
TC: total cholesterol
TG: total triglycerides
LDL-C: low-density lipoprotein cholesterol
PBS: phosphate-buffered saline
GAPDH: glyceraldehyde-3-phosphate dehydrogenase
ROC: receiver operating characteristic
OR: odds ratio
AUC: area under the curve
CTSS: cathepsin S
